# Focal Laser Ablation of Prostate Cancer: Numerical Simulation of Temperature and Damage Distribution

**DOI:** 10.1186/1475-925X-10-45

**Published:** 2011-06-02

**Authors:** Mohamad-Feras Marqa, Pierre Colin, Pierre Nevoux, Serge R Mordon, Nacim Betrouni

**Affiliations:** 1 Inserm (French National Institute of Health and Medical Research), U703, 152 rue du Docteur Yersin, 59120 Loos, France; 2 Université Lille Nord de France, F-59000 Lille, France; 3 CHU Lille, F-59000 Lille, France

**Keywords:** Prostate cancer, focal laser ablation, thermal damage, bioheat transfer simulation

## Abstract

**Background:**

The use of minimally invasive ablative techniques in the management of patients with low grade and localized prostate tumours could represent a treatment option between active surveillance and radical therapy. Focal laser ablation (FLA) could be one of these treatment modalities. Dosimetry planning and conformation of the treated area to the tumor remain major issues, especially when, several fibers are required. An effective method to perform pre-treatment planning of this therapy is computer simulation. In this study we present an *in vivo *validation of a mathematical model.

**Methods:**

The simulation model is based on finite elements method (FEM) to solve the bio-heat and the thermal damage equations. Laser irradiation was performed with a 980 nm laser diode system (5 W, 75 s). Light was transmitted using a cylindrical diffusing fiber inserted inside a preclinical animal prostate cancer model induced in Copenhagen rats. Non-enhanced T2-weighted and dynamic gadolinium-enhanced T1-weighted MR imaging examinations were performed at baseline and 48 hours after the procedure. The model was validated by comparing the simulated necrosis volume to the results obtained *in vivo *on (MRI) and by histological analysis. 3 iso-damage temperatures were considered 43° C, 45° C and 50° C.

**Results:**

The mean volume of the tissue necrosis, estimated from the histological analyses was 0.974 ± 0.059 cc and 0.98 ± 0.052 cc on the 48 h MR images. For the simulation model, volumes were: 1.38 cc when T = 43° C, 1.1 cc for T = 45°C and 0.99 cc when T = 50 C°.

**Conclusions:**

In this study, a clear correlation was established between simulation and *in vivo *experiments of FLA for prostate cancer.

Simulation is a promising planning technique for this therapy. It needs further more evaluation to allow to FLA to become a widely applied surgical method.

## Background

Natural history of prostate cancer (PCa) is characterized by the frequent multifocality of the pathology. Cancer multifocality has been reported in 50 to 87% in contemporary series of radical prostatectomies [1]. However, previous studies ([2], [3], [4]) have shown that in case of multifocality localization, only the 'index' (i.e. the principal focus) lesion volume is a predictive factor of progression. A 0.5 cc volume threshold is currently accepted to define a lesion with clinically-significant size. This volume is associated with a 10% risk of extra-capsular extension and metastasis [5].

Current therapeutic recommendations for localized prostate cancer consist in radical options aiming to treat the prostatic gland in its totality (radical prostatectomy, radiotherapy, brachytherapy, or HIFU). The use of minimally invasive ablative techniques in the management of patients with low grade and localized tumours could represent a treatment option between active surveillance and radical therapy. Focal laser ablation (FLA) could be one of these treatment modalities. FLA is under development as a minimally-invasive technique for *in situ *destruction of solid-organ tumours. Based on the use of low-power laser, which delivers luminous energy using an adapted optical system, FLA produces a coagulation necrosis zone, which volume can be controlled, reducing the risk of damage to healthy adjacent structures.

First results of the use of FLA as management option for men with low risk PCa were reported by the Toronto team ([6], [7]). Technical feasibility of the method was proved and phase 1 clinical trial was conducted on 12 patients. The authors reported that biopsies at 6 months in 67% patients were negative in the treated area.

The biological effects of laser energy depend on the laser wavelength, laser power, the pulse duration, blood perfusion and both the optical and thermal properties of the tissue involved [8]. Therefore, treatment monitoring is required in order to have precise information about the extent of thermal damage in tissues caused by laser interstitial coagulation. Modeling laser-tissue interaction is a potent tool to help analyzing and optimizing the parameters governing planned laser surgical procedures. Nevertheless, an adequate model with adequate accuracy remains to be developed.

Most suggested models depend on a large extent of simplifications of the real problem, either in the geometry they offer or in the system of equations they use. Roggan *et al. *[8] used Monte-Carlo (MC) simulation to simulate the use of multiple applicators, but this method is limited to symmetrical geometries and has not been correlated to real anatomic datasets. A similar study using finite difference method (FDM) to describe laser-tissue interaction was proposed by Whelan *et al. *[9], but the authors did not include the coagulation process with its irreversible changes in thermal tissues properties.

Some models used the bioheat equation and considered the role of the changes in the tissue properties during temperature elevation processes. However, these models presented deviations from the experimental results because of using inaccurate optical tissues properties. Mohammed et Verhey [10] proposed a method based on finite element modeling (FEM) and the combination of light and bioheat equations to determine the heat extent and damage distribution near main vessels. The authors checked their model during *in vitro *experiments. The deviation between their model and the experiment were 5% in x-direction and 20% in y-direction. They considered that the main reasons behind this deviations lies with: 1) the use of inaccurate values for the optical tissues properties, 2) The available memory limits which affect the performance of the machine calculation, and 3) the absolute tolerance used in the solver, where they used an absolute tolerance value of 0.01.

Very few modeling methods have simulated the behavior of *in vivo *laser-tissue interaction. The aim of this paper is to validate a 3D simulation model for calculating the heat extent and estimating the volume of damaged tissue. Ground truth for the validation was available from the *in vivo *prostate cancer pre-clinical model.

## Materials and methods

The model used to simulate focal laser-ablation (FLA) in *in vivo *prostate tissues uses the FEM method to solve the bio-heat equation. Thermal prostate tissues properties were reported from literature ([11], [12]).

### Preclinical model

Investigations were conducted in accordance with accepted ethical and human practices, and approved by the local animal care committee at our institution. (Ethic Committee in Animal Experimentation of Lille University; agreement number: A59-35010 DHURE, file number:CEEA - 14-2009).

Preclinical model consisted in Dunning R3327-AT2 syngenic prostate adenocarcinoma implanted (2 × 10^6 ^cells) by subcutaneous injection in the flank of Copenhagen rat, 8 weeks of age or older (Harlan Laboratories TM). Ten rats were used in this study.

### Experimental set up

Laser delivery was performed with a commercially available diode laser unit (Pharaon 980, Osyris, Hellemmes, France). This system delivers a maximum output of 15 W in a CW or pulsed mode, at 980 nm. Laser light was transmitted through cylindrical diffusing fiber (CDF) of 10 mm length with a 500 μm core diameter.

**Figure 1:**
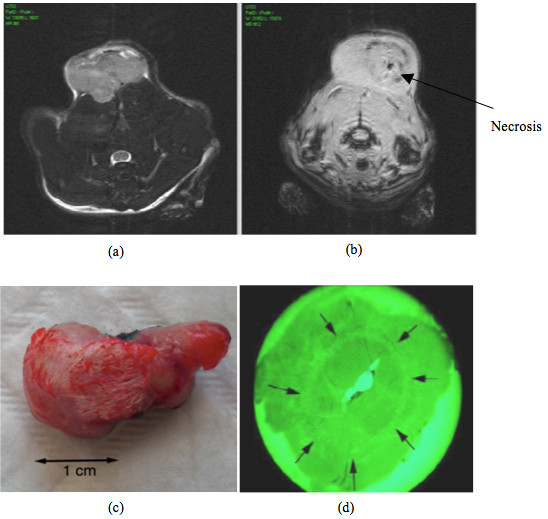
**(a) Pre-treatment MR image of the rat**. (b) Post-treatment MR image with the visualization of the necrosis. The image is in a different plane than image (a). (c) The tumor after treatment and excision. (d) Histological tumor slice with green filter to enhance the circular limits of the coagolative necrosis (black arrows).

One hour before the procedure, a multi-spectral Magnetic Resonance Imaging (MRI) acquisition was performed on the animals using a 7 Tesla MRI unit (Biospec, Bruker BioSpin SA, USA). Acquisitions included T2 weighted (T2W) images and dynamic contrast enhanced T1 (DCE) images. The MR images were used for the pre-treatment planning of the procedure by defining the fiber trajectory (Figure 1.a). The axis and depth of optimal fiber implantation were spotted on these image sequences. The purpose of this identification was to avoid fiber implantation in a spontaneously necrotic area.

In order to induce local hyperthermia in the sub-cutaneous tumor, the laser procedure was performed as follows: the CDF was inserted into the center of the tumor as planned on the pre-treatment MRI. The power provided from the source was 5 Watt with energy fluence of 1145 J/cm^2^. The irradiance duration was 75 s. Measured initial temperature of tissue was T_0 _= 37 ± 0.5°C. A thermocouple was used to measure the maximum temperature at the tip end of CDF which was (91 ± 1) C°.

For the final positioning of the fiber, ultrasound imaging (US) was used to monitor the insertion according to the MR planned trajectory. The distance between the skin entry port and the end of the fiber diffusing part was measured on the US images. This distance corresponded to the implantation depth measured on MRI.

48 hour after the procedure, another MR imaging including the same sequences (T2W and DCE) were acquired. After this acquisition, the animals were euthanized and tumors were removed. Histological analyses were performed and induced necrosis volume measured (Figure 1.b, Figure 1.c and Figure 1.d).

### Modeling

In the following sections, we will describe the different stages to construct the model:

### Geometrical model

Starting from the MR images, a region of interest (ROI) was defined, enclosing both the tumor and the fiber. We used the dimensions of the field of view of the MR images to define the dimensions of the geometrical model. It consisted of cube of 70 × 70 × 20 mm^3^. Then, the fiber trajectory seen on the MR images was used to define and to simulate the fiber in the model. Tissue around the fiber in the model was considered homogenous.

### Heat distribution

The absorption of light in tissue causes a local elevation in temperature. Tissue heat transfer due to the energy of light deposited is described by the well known bioheat transfer equation (Pennes equation):(1)

Where T is temperature (°K)

C_p _= C*ρ is heat capacity (J.mm^-3^.°K^-1^),

ρ is tissue density (g.mm^-3^),

C is specific heat capacity of tissue (J.g^-1^.°K^-1^),

*k *is thermal conductivity of tissue (W.mm^-1^.°K^-1^),

w_b _is blood flow rate (ml.g^-1^.min^-1^),

T_b _is the blood temperature,

t is time (s),

Q_abs _is the heat source (W.mm^-3^),

Q_met _is the metabolic heat source (W.mm^-3^).

The precise evaluation of the optical parameters of prostate tissue remains a challenging issue and all reported studies confirmed that they are heterogeneous and their integration in a modeling process remains difficult and imprecise. For these reasons we considered that the

Q_abs _term corresponds to the heat source and a constant value of 5 W.mm^-3 ^was fixed in the model.

For the numerical application of these parameters for the pre-clinical model, we used the values reported in [11]. They are summarized in table 1.

**Table 1:** Physical parameters of the AT-1 Dunning rat prostate used in the numerical simulation extracted from reference [11] and reference [12] and corresponding to the wave length λ = 980 nm.

Parameter	Value
Specific heat capacity, C (J.g^-1^.°K^-1^)	4.20
Density, ρ (g.mm^-3^)	0.999 × 10^-3^
Thermal conductivity, *k *(W.mm^-1^.°K^-1^)	5.52 × 10^-4^
Blood flow rate, wb (ml.g^-1^.min^-1^)	0.10
Frequency factor, Af (s^-1^)	3.8 × 10^14^
Activation energy, Ea (J.mole^-1^)	1.084 × 10^5^
Universal gas constant, R (J.mole^-1^.°K^-1^)	3.14847

From the literature, in all cases the effects of metabolic heat source was considered insignificant [13].

FLA model is based on the finite element method (FEM). The geometry of the source was incorporated in the model as following: the starting point for the finite element method is a mesh, which consists in a partition of the geometry into small units of a simple shape (triangles or squares), called mesh elements (nodes).

The simulated laser source was considered as a cylinder, which has the same dimensions of the source of laser used in the experiments.

The tissues were considered as a regular finite element grid of 70 × 70 × 20 mm^3 ^corresponding to the MR images. The initial temperature was set to 37°C.

The boundary conditions for the bioheat equation were:

T = T_b _for the cylindrical wall,

 for all other surfaces.

Where  is the direction of the heat flux.

To obtain a stable and convergent numerical solution, the GMRES (Generalized Minimum RESidual) algorithm was used. GMRES is an iterative method introduced by Saad and Schultz [14] to solve system of linear equations. It was tuned with the following settings: time steps were 0.5 s and convergence tolerance was set to 10^-3^.

### Thermal damage

Thermal damage in cells and tissue can be described mathematically by a first-order thermal-chemical rate equation, in which temperature history determines damage. Damage is considered to be a uni-molecular process, where native molecules are transformed into a denatured/coagulated state through an activated state leading to cell death. Damage is quantified using a single parameter Ω, which ranges on the entire positive real axis. It is calculated from the Arrhenius law ([15], [16]). Ω is dimensionless, exponentially dependent on temperature and on time of exposure. It is calculated from the Arrhenius law as:(2)

where C_(r,0)_, C_(r,τ) _are the concentrations of the undamaged molecules at the beginning and at time τ, respectively.

A_f _(s^-1^**) **is the frequency factor,

E_a _(J.mole^-1^) is the activation energy,

R (J.mole^-1^.°K ^-1^) is the universal gas constant, and

T(°K) is the temperature

The parameters: A_f_, E_a_, called the kinetic parameters, are temperature dependent and can be determined by the experimental. Numerical values of these parameters for the Dunning R3327-AT prostate tissues, which correspond to temperatures measured at the tip end of the CDF, were reported in [12] (table 1).

Equation 2 indicates that the measure of damage (Ω) describes the probability of tissue being destroyed. It is the logarithm of the ratio of the initial concentration of undamaged tissue to the concentration once damage has accumulated, for the time interval t = 0 to t=τ. Therefore, Ω = 1 corresponds to an irreversible damage of 100% of the affected cells.

The damage threshold for tissue necrosis is commonly selected as omega = 1 (a damage concentration of 63% for a unimolecular system). When performing an Arrhenius analysis, omega > 1 is assumed to correspond with an experimental endpoint- typically a visible increase in light scattering, which makes the tissue appear whiter than the necrotic tissue. The necrosis border corresponds to Ω = 1.

### Model validation

In a previous study [17], we demonstrated the correlation (Pearson correlation index r = 0.87) between MR imaging measurements of necrosis induced by interstitial laser and histology measurements. In fact, the histological analysis of thermal damage after 48 h displayed the same ellipsoid shape as the one observed on MRI. Thus, to validate the proposed simulation model, we compared the simulated results with the MRI and histology observations.

Hyperthermia literature often cites 43 degrees Celsius (43° C) as the point at which thermal damage occurs to tissues [18] but this value depends on the energy source. For instance, with radiofrequency ablation, a temperature of 47° C is generally accepted [13].

In this study, 3 temperatures were considered: 43°C, 45°C and 50°C and the volume of simulated isotherm for each temperature was compared to the MRI and macroscopic volumes.

### Numerical implementation

The mathematical model was implemented using the COMSOL MULTIPHYSICS V4.0 (COMSOL Inc., Palo Alto, USA) software. This Finite Element computer aided design software specifies the Partial Differential Equations, variables, geometry and boundary conditions.

## Results

**Figure 2:**
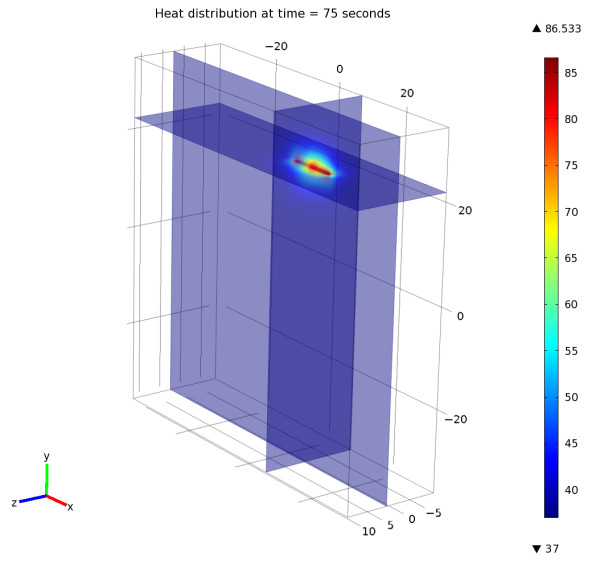
**The solution of the heat distribution equation at the time = 75 seconds**. The solution is elliptical and corresponds to the length of the diffusing tip of the fiber. This figure is available as a video. The video demonstrates the temperature rise inside the tissues (Additional file 1).

Figure 2 illustrates the resolution of the bioheat equation with a temperature map at the end of laser irradiation (t = 75 s) time. The solution shape is elliptical with a principal axis corresponding to the length of the diffusing tip. Figure 2 is available as a video; this video demonstrates the temperature rise inside the tissues. (Additional file 1)

Table 2 presents the necrosis volumes for ten rats calculated on MR images and from the histological analysis after 48 hours from the laser procedure. The mean volume of the tissue necrosis, estimated from the histological analyses was 0.974 ± 0.059 cc and 0.98 ± 0.052 on the 48 h MR images respectively. For the simulation model, volumes were: 1.38 cc when T = 43° C, 1.1 cc for T = 45°C and 0.99 cc when T = 50 C°.

**Table 2:** The necrosis volumes for ten rats calculated on the MR images and from the histological analysis.

Rat	Necrosis volume on MRI at 48 h (cm^3^)	Histological necrosis volume (cm^3^)
Rat # 1	0.953	0.945
Rat # 2	0.970	0.950
Rat # 3	0.987	0.923
Rat # 4	0.946	0.990
Rat # 5	0.933	0.923
Rat # 6	0.967	0.941
Rat # 7	0.978	0.992
Rat # 8	0.989	0.988
Rat # 9	0.958	0.967
Rat # 10	1.121	1.125

Mean volume (cm^3^)	0.98 ± 0.052	0.973 ± 0.059

The Pearson correlation index was r = 0.87 between the pathology volumes and the MRI volumes.

**Figure 3:**
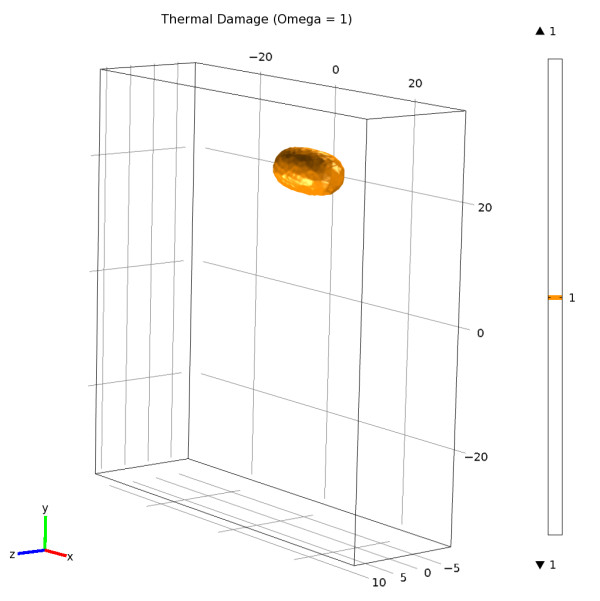
**Thermal damage in tissues resulting from the simulation and corresponding to (Ω = 1)**. The thermal damage is available as a video stream. This video shows how the thermal damage occurs and growing with the time around the laser fiber (Additional file 2).

Figure 3 depicts the thermal damage in tissues resulting from the simulation and corresponding to (Ω = 1). Figure 3 is available as a video; this video shows how thermal damage occurs and grows in tissues in time around the laser fiber (Additional file 2).

**Figure 4:**
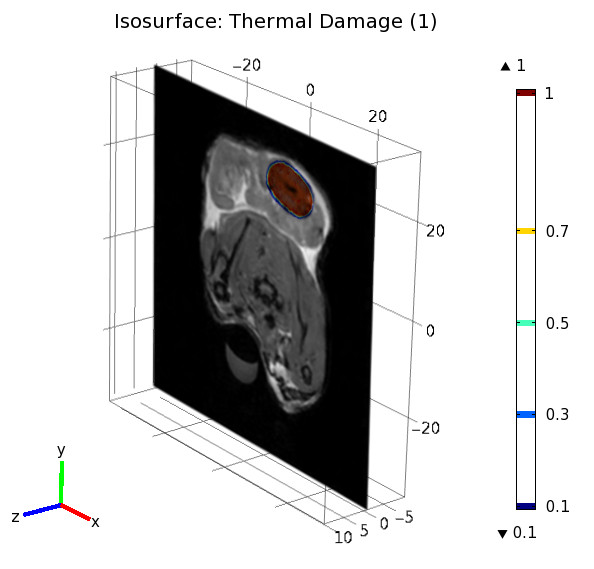
**Iso-surface of the thermal damage matched with the 48 hours MR image**. More details about the form of the thermal damage are available as a video stream. This video demonstrates the form of the thermal damage around laser fiber and how this damage appears inside tissues (Additional file 3).

At the 48 h MRI control, the necrosis edges were visible in T1-weighted Turbo FLASH sequence without gadolinium enhancement. These limits corresponded to an hypo-intensive border. Lesions induced by FLA have a prolate spheroid shape. Figure 4 shows the simulated necrosis corresponding to the iso-damage Ω = 1. The necrosis is merged with the 48 h MR image to highlight the correlation between the two results.

More details about the form of the thermal damage are available in as a video. This video demonstrates the form of the thermal damage around laser fiber and how this damage appears inside tissues (see Additional file 3).

## Discussion

The concept of partial treatment or focal therapy for prostate cancer is recent and controversial in the urological community owing to the frequency of tumor multifocality. However, in selected patients, this option could be an intersecting alternative for low risk prostate cancers. Before the generalization of this concept, many issues have to be addressed. First, accurate localization of the tumor is required. For this purpose, ongoing work on the diagnosis and staging of tumors using multimodality imaging (ultrasound, elastography, multiparametric MR,..) must be performed ([19], [20]). The second issue is the treatment planning required to optimize therapy parameters to ensure the optimal coverage of the area while sparing surrounding tissue. This issue is challenging and still needs the development of dedicated dosimetric tools as it was the case for radiotherapy and brachytherapy.

Indeed, in the first clinical trials of focal laser ablation of PCa [21], the authors reported the following energies: 3260 J, 4014 J, 3516 J and 5900 J to obtain ablated volumes: 4.5 cm^3^, 2.8 cm^3^, 2 and 3.5 cm^3 ^respectively. This represents energy volume ratios of 724 J/cm^3^, 1486 J/cm^3^, 1406 J/cm^3 ^and 1311 J/cm^3 ^respectively. This important variability could be explained by an overtreatment of some areas.

In a previous study for the establishment of an FLA protocol for prostate cancer, where experiments were conducted on Dunning R3327-AT2 rat, we have obtained robust parameters of power (5W) and time (75 s) to obtain a reproducible necrosis of 1 cm^3 ^[16]. In this study, we were interested in the simulation of the FLA outcomes by *in vivo *validation of a theoretical model. This generic model was already described and used for different cancers. We have applied it to the pre-clinical model by defining the accurate physical parameters.

The simulations were realized by the resolution of the heat diffusing equation and by the modeling of thermal damage Ω. In this model, solving the Arrhenius equation for time increasing temperature returns a relatively constant damage threshold value of 50 ± 1°C for t = 75 s exposure (Figures 2 and 3). Although this model greatly simplifies the understanding of thermal tissue damage by assuming a single first-order rate process and to not directly modeling the energy of the laser light, it has been successfully used to describe the threshold of tissue damage as a function of temperature and exposure time.

Previous theoretical models of prostate treatment have generally assumed threshold damage temperatures of 50°C. These values are based on studies involving exposure durations about seconds or greater. For instance, histological evaluation performed by Peters *et al*. showed that the thermal-injury boundary can be predicted from a threshold-maximum temperature of approximately 51 degrees C° or an equivalent Arrhenius t(43) period of 200 minutes ([22], [23], [24]).

One limit of the current study stems from local tissue heterogeneities, uncertainties in optical and thermal properties and blood perfusion rate in human prostate. These heterogeneities observed from patient-to-patient and from base-to-apex of the prostate complicate the prediction of FLA thermal damage. The last issue consisting in the treatment monitoring could manage this limitation. The procedure could be guided by images: temperature sensitive MR sequences or contrast-enhanced ultrasound elastography. These control methods can either measure the temperature [25], the heat distribution or the tissue perfusion, but do not define the final necrosis. In fact, in thermotherapy, the heat continues to spread after irradiation so that it is nearly impossible to accurately identify the actually treated volume, without waiting several hours after the end of the treatment.

On the other hand, good pre-treatment planning does not guarantee a good treatment. Indeed, the treatment is correctly achieved when the fibers are inserted in the right positions and in the right directions. The shape of prostate is different between the planning MR images and the intra-treatment guidance ultrasound images. This fact makes difficult the correct mapping of the MR planned positions by the sole ultrasound guidance. Combination of these two modalities could be a solution to enhance the accuracy of fibers insertion. Currently we are working on the integration of these techniques in the treatment planning software to ensure the correct mapping of the optimized positions.

## Conclusion

Focal laser ablation of PCa is a promising therapy technique. It needs further more evaluation and understanding of the heat extent in tissues to become a surgical method applied in the routine hospitalization. In this paper we presented a numerical simulation model of FLA and we validated it these simulations with *in-vivo *experimental results conducted on Dunning R3327AT-2 rat. A laser diode system attached to a cylindrical diffusing fiber (CDF) was used to diffuse laser at 980 nm wavelength at power 5 W during 75 seconds in tissues.

This approach could be a first step for a greater understanding of global impact of laser-tissue interaction through the calculation of heat distribution and the thermal damage. Post-laser thermotherapy tissue injury was quantified by calculating the thermal damage (Ω). The threshold of irreversible cellular injury where Ω = 1 corresponding to a temperature of 50°C.

## Competing interests

The authors declare that they have no competing interests.

## Authors' contributions

MFM: Carried out the numerical simulation and helped to draft the manuscript. PC: performed the animal experimentations and discussed the clinical data. PN: Participated to the animal experimentations. SRM: Brought the main idea of the work and helped in designing the study. NB: Designed the study and drafted the manuscript. All authors read and approved the final manuscript

## Supplementary Material

Additional file 1 **Video 1**. This video demonstrates the temperature rise inside the tissues and shows how the heat distribution in tissues appears. Click here for file

Additional file 2 **Video 2**. This video shows how the thermal damage occurs and grows in time around the laser fiber. Click here for file

Additional file 3 **Video 3**. This video stream demonstrates the form of the thermal damage around the laser diffuser and how this damage appears inside tissues. Click here for file
