# Supplementary figures and images for: Focal Laser Ablation of Prostate Cancer: Numerical Simulation of Temperature and Damage Distribution
Source: Biomed Eng Online. 2011 Jun 2;10:45. doi: 10.1186/1475-925X-10-45 (PMC3117748; doi:10.1186/1475-925X-10-45)

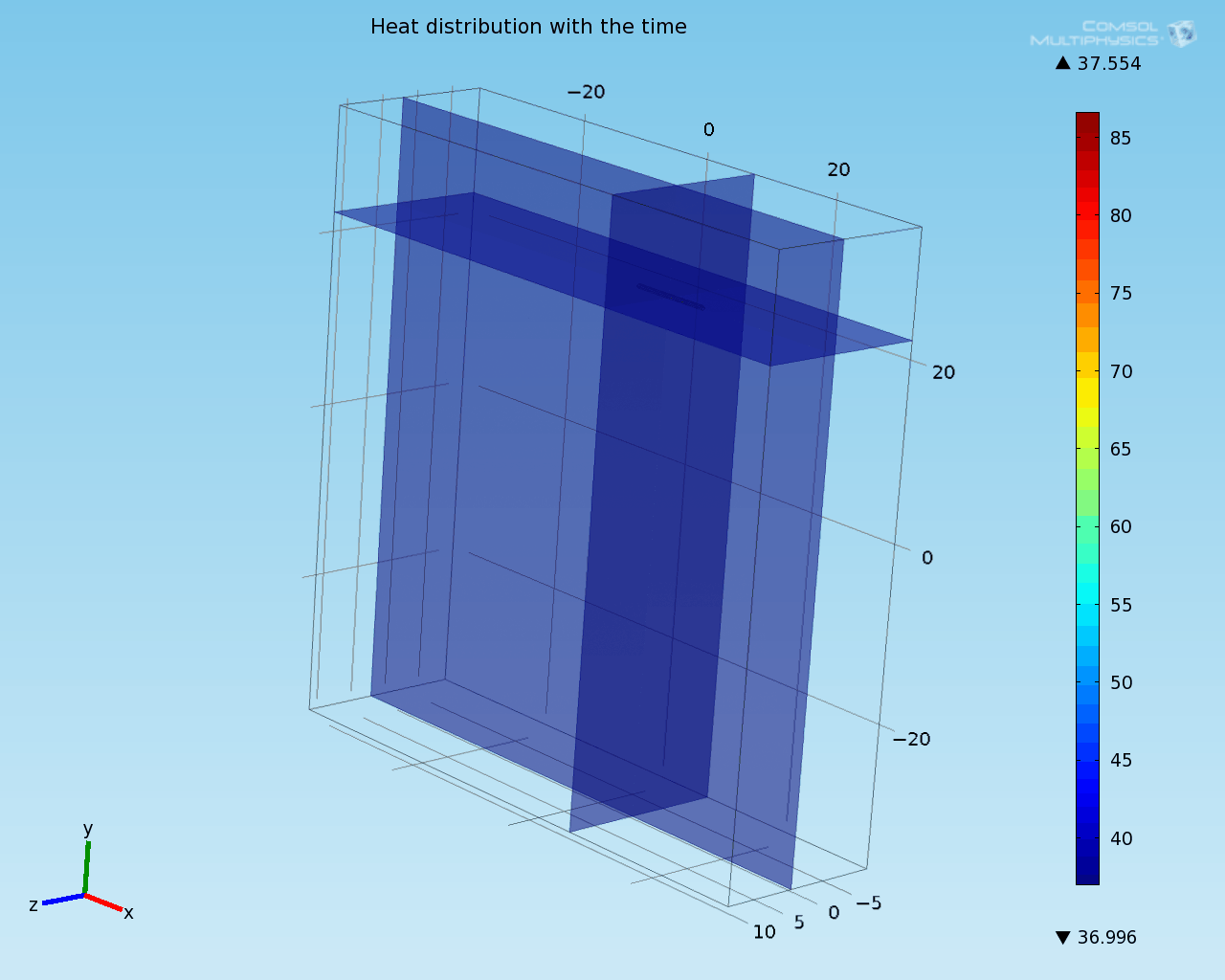

Supplement: Additional file 1 — Video 1. This video demonstrates the temperature rise inside the tissues and shows how the heat distribution in tissues appears. [file 1475-925X-10-45-S1.GIF]

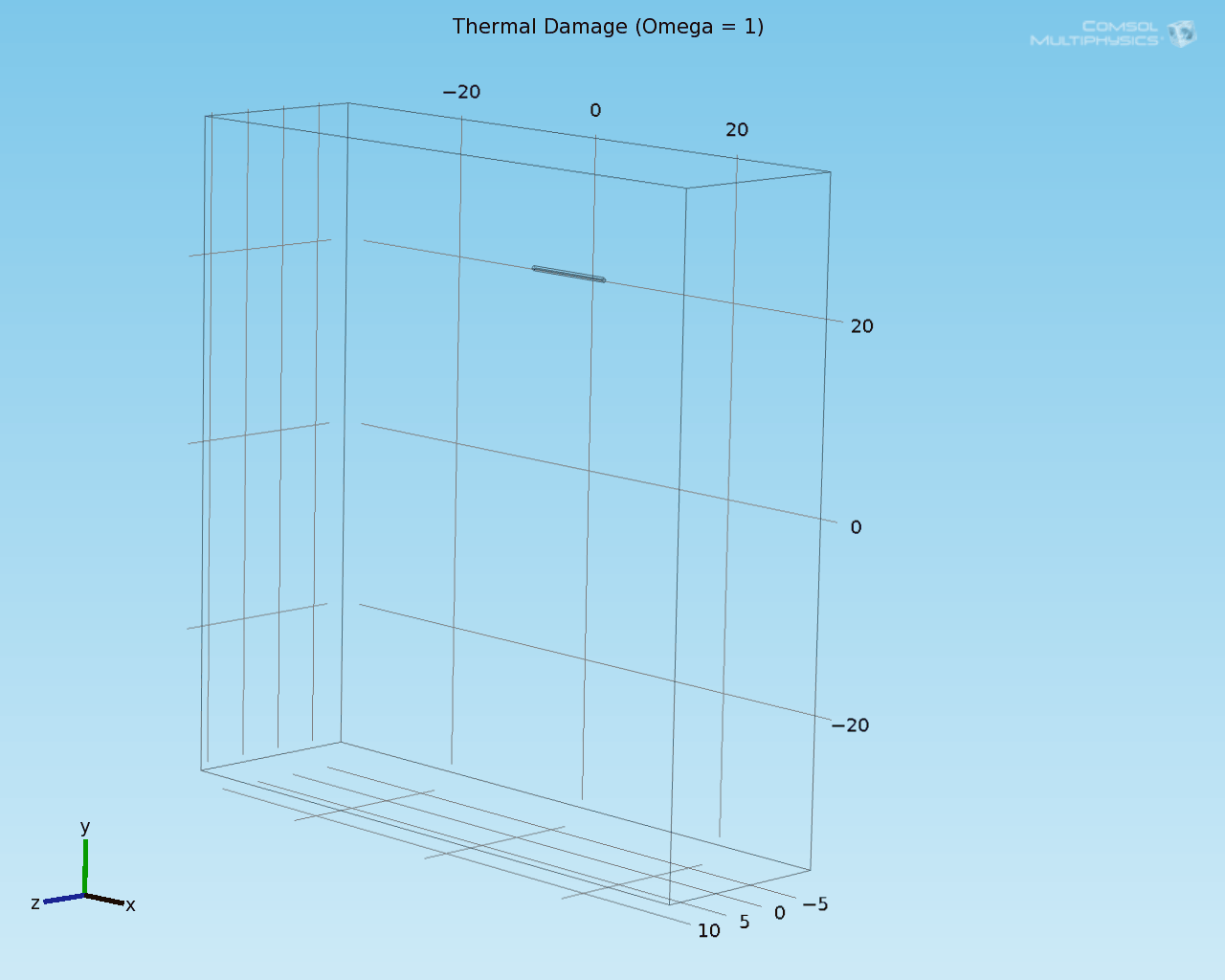

Supplement: Additional file 2 — Video 2. This video shows how the thermal damage occurs and grows in time around the laser fiber. [file 1475-925X-10-45-S2.GIF]
